# Pure Polycyclic Aromatic Hydrocarbon Isomerides with Delayed Fluorescence and Anti‐Kasha Emission: High‐Efficiency Non‐Doped Fluorescence OLEDs

**DOI:** 10.1002/advs.202304204

**Published:** 2023-09-17

**Authors:** Haoxin Huang, Nengquan Li, Shuguang Fu, Xuechao Mo, Xiaosong Cao, Xiaojun Yin, Chuluo Yang

**Affiliations:** ^1^ Shenzhen Key Laboratory of New Information Display and Storage Materials College of Materials Science and Engineering Shenzhen University Shenzhen 518060 P. R. China

**Keywords:** anti‐Kasha emission, full‐color emission, organic light emitting diodes, pure hydrocarbon, thermally activated delayed fluorescence

## Abstract

Pure polycyclic aromatic hydrocarbons (PAHs) consisting solely of carbon‐hydrogen or carbon‐carbon bonds offer great potential for constructing durable and cost‐effective emitters in organic electroluminescence devices. However, achieving versatile fluorescence characteristics in pure PAHs remains a considerable challenge, particularly without the inclusion of heteroatoms. Herein, an efficient approach is presented that involves incorporating non‐six‐membered rings into classical pyrene isomerides, enabling simultaneous achievement of full‐color emission, delayed fluorescence, and anti‐Kasha emission. Theoretical calculations reveal that the intensity and distribution of aromaticity/anti‐aromaticity in both ground and excited states play a crucial role in determining the excited levels and fluorescence yields. Transient fluorescence measurements confirm the existence of thermally activated delayed fluorescence in pure PAHs. By utilizing these PAHs as emitting layers, electroluminescent spectra covering the entire visible region along with a maximum external quantum efficiency of 9.1% can be achieved, leading to the most exceptional results among non‐doped pure hydrocarbon‐based devices.

## Graphical abstract

The incorporation of non‐six‐membered rings into pure polycyclic aromatic hydrocarbon (PAH) isomerides offers a new concept to consciously reconstruct their frontier molecular orbitals, thus enabling the simultaneous realization of full‐color emission, delayed fluorescence, and anti‐Kasha emission. Non‐doped PAH‐based electroluminescent devices achieve a high *η*_ext,max_ of 9.1%.

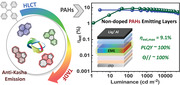

## 1 Introduction

**Figure 1:**
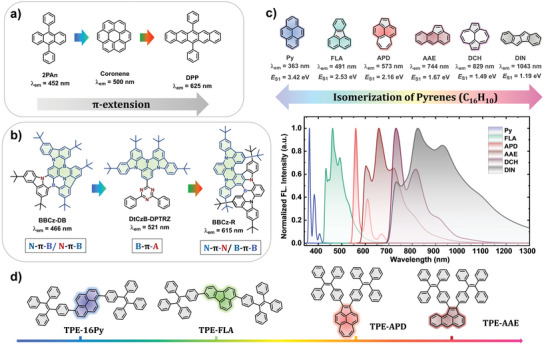
Typical strategies to realize full‐color emission in OLEDs involve a) π‐extension or b) incorporating heteroatoms at specific position. c) Modeling the combination of molecular orbitals in simple pyrene isomers offers distinct emission spectra cover from the near ultraviolet band to near‐infrared band (simulated under vacuum condition). d) Chemical structures of these pyrene isomers‐cored emitters equipped with TPE units at peripheral.

Polycyclic aromatic hydrocarbons (PAHs) devoid of heteroatom incorporation have garnered substantial attention within the realm of organic functional semiconductors due to their potential in fabricating robust materials with durability and cost‐effectiveness.^[^ ^1^ ^]^ However, the intrinsic nature of pure PAHs, consisting solely of carbon‐hydrogen/‐carbon bonds, offers limited avenues for modulating their emission bands, frontier molecular orbitals, and associated energy levels.^[^ ^2^ ^]^ To achieve full‐color emission in pure PAHs, extending the π‐delocalization from oligocyclic to fused polycyclic arenes serves as the primary guidance (**Figure** **1**a).^[^ ^3^ ^]^ Nonetheless, this approach often leads to aggregation‐caused quenching (ACQ) and poor processibility, which significantly hinder their application in organic light‐emitting diodes (OLEDs). In contrast, the introduction of heteroatoms with distinct electronegativities into the PAH framework provides versatile and convenient avenues for developing desired organic emitters.^[^ ^4^ ^]^ For example, embedding electron‐deficient boron and electron‐rich nitrogen at specific positions within the PAH backbone (Figure 1b) represents a well‐established strategy for simultaneously achieving full‐color, narrowband emission and a small energy difference (Δ*E*_ST_) between the lowest singlet (*S*_1_) and triplet (*T*_1_) states, commonly referred to as multiple resonance thermally activated delayed fluorescence (MR‐TADF).^[^ ^5^ ^]^ However, this approach comes with associated drawbacks, including complex synthesis processes, inferior chemical stability, and rapid device efficiency roll‐offs at high luminance.^[^ ^6^ ^]^ As a result, reevaluating heteroatom‐free PAHs may provide an alternative opportunity.

In recent years, there has been significant expansion in the development of pure PAHs,^[^ ^7^ ^]^ following the pioneering work involving single crystal anthracene in electroluminescence devices.^[^ ^8^ ^]^ Particularly noteworthy are the advancements in pure PAHs exhibiting high *T*_1_ levels and balanced hole/electron transport, positioning them as promising hosts for the phosphorescent OLEDs.^[^ ^1d^ ^]^ Remarkably, the optimized external quantum efficiencies (*η*_ext_) achieved in blue, green, red, and white devices have already surpassed 25%.^[^ ^1b,c^ ^]^ To address potential instability concerns in OLEDs, it is preferable to utilize emitting layers that consist of pure PAHs. However, a major challenge lies in the limited tunability of key photophysical properties within hydrocarbon‐based materials,^[^ ^9^ ^]^ resulting in the current lack of focus on the emitters themselves. In addition, the incorporation of bulky steric hindrance groups, such as tetraphenyl ethylene (TPE) or adamantane, presents a universally applicable method to mitigate the ACQ effect in planar emitters.^[^ ^10^ ^]^ In light of these considerations, the amalgamation of a versatile PAH core with steric groups at the periphery represents a promising avenue for achieving non‐doped pure hydrocarbon‐emitting layers.

In this work, we present a novel conceptual framework by incorporating non‐six‐membered rings into pyrene isomerides to consciously modify the arrangement of frontier molecular orbitals,^[^ ^11^ ^]^ and therefore affording versatile PAH emitters suitable for OLED applications. Notably, the calculated singlet excited energy levels (*E*_S1_) show wide‐range tunability ranging from 3.42 eV (pyrene, Py, ultraviolet emission) to 1.19 eV (indeno[2,1‐*a*]indene, DIN, near‐infrared emission) within the same C_16_H_10_ isomer family (Figure 1c). Accordingly, the 2D iso‐chemical shielding surface (2D‐ICSS) and hole‐electron analysis clearly demonstrated that the variation of aromaticity/anti‐aromaticity between ground state and corresponding excited states primarily determine their energy gaps. Interestingly, we also observe anti‐Kasha emission from the aceanthrylene (AAE) core, which can be attributed to an increased Huang‐Rhys factors (HRFs) at both high‐ and low‐frequency vibrational modes, a higher internal conversion rate (*k*_ic_), and a lower radiation transition rate (*k*_r_) of *S*_1_→*S*_0_ than *S*_2_→*S*_0_. In the pursuit of non‐doped pure hydrocarbon emitting layers, four carefully selected Py isomerides incorporating neutral TPE were effectively synthesized as an illustrative example (Figure 1d), resulting in distinct electroluminescence spectra upon utilization as emitters in OLEDs. Particularly noteworthy, TPE‐APD exhibited a characteristic thermally activated delayed fluorescence (TADF) feature, both in dilute toluene solution or 2,6‐bis[3‐(9*H*‐carbazol‐9‐yl)phenyl]pyridine (26DCzPPy) matrix. Consequently, this study presented the first instance of TADF‐based OLEDs utilizing pure hydrocarbon emitters. Furthermore, the devices utilizing both TPE‐16Py and TPE‐FLA as emitting layers exhibit notably high maximum external quantum efficiencies (*η*_ext,max_) of 7.2% and 9.1%, respectively. These values surpass the theoretical threshold of 5% for conventional fluorescence‐based OLEDs, thus exemplifying the exceptional performance achieved by pure hydrocarbon‐based non‐doped devices.^[^ ^10b^ ^]^

## 2 Results and Discussion

Initially, theoretical calculations were performed to learn the diversity of molecular orbitals among different Py isomerides,^[^ ^12^ ^]^ and the results were listed in Table S1 (Supporting Information). It is noteworthy that as the fused non‐six‐membered rings varied from fluoranthene (FLA) to acepleiadylene (APD), AAE, and then DIN, both the energy gaps and the LUMOs exhibited a gradual decrease. Furthermore, the shape and characteristics of the MOs exhibited distinct variations among different pyrene isomers, leading to diverse transition models. For instance, the spatial distribution of LUMO+1, LUMO, HOMO, and HOMO‐1 covered the entire Py skeleton uniformly, indicating localized electronic transitions between the occupied π orbitals and the unoccupied π* orbitals. Conversely, in the case of AAE, the distributions of HOMO and HOMO‐1 were predominantly localized in a specific region of the fused ring, which endowed it with a property of partial charge‐transfer transition and consequently resulted in a lower *S*_1_ energy level.^[^ ^13^ ^]^

**Figure 2:**
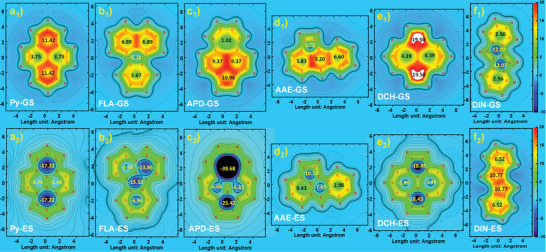
Calculated 2D‐ICSS(1)zz maps (1 Å above the XY planes) of these pyrene isomers, a_1_)/a_2_) Py, b_1_)/b_2_) FLA, c_1_)/c_2_) APD, d_1_)/d_2_) AAE, e_1_)/e_2_) DCH, and f_1_)/f_2_) DIN. The upper row of a_1_)–f_1_), and the lower row of a_2_)–f_2_) display the results of ground and excited states respectively.

The pure PAHs were subjected to nucleus‐independent chemical shift (NICS) and 2D‐ICSS(1)zz calculations at the B972/def2TZVP level, revealing alterations in aromaticity/anti‐aromaticity between the ground state (GS) and excited state (ES). Notably, due to the presence of strong spin‐spin interactions, the ES discussion focused on the *T*_1_ state rather than the *S*_1_ state in here. **Figure** **2**a_1_,a_2_ illustrated that the bilateral benzene rings along the long axis of the Py core exhibited pronounced aromaticity in the GS state, but transition to strong anti‐aromaticity in the ES state. This indicated significant differences in electronic structures between the ground and excited states and leading to high excitation energy accordingly. With the incorporation of antiaromatic cyclopentadiene (CP) moiety, the trends of aromaticity were gradually decreasing or even reverse (Figure 2b_1_/b_2_–2f_1_/f_2_). For instance, in the case of DIN, the central pentalene fragment exhibited strong anti‐aromaticity in the GS state, but undergone a notable transition to powerful aromaticity in the ES state, resulting in a significant reduction in the excitation energy.^[^ ^14^ ^]^ However, this pattern does not hold true for APD and dicyclopenta[*ef*,*kl*]heptalene (DCH), which might be attributed to the containing of characteristic moiety similar to “azulene” (5π+7π), and affording strong aromatic property at GS.^[^ ^15^ ^]^ In addition, similar tendencies can be observed when incorporating neutral TPEs at the peripheral of these C_16_H_10_ isomerides, which was presumably due to the involved transitions largely relying on the central PAH cores (Figure S8, Supporting Information).

**Figure 3:**
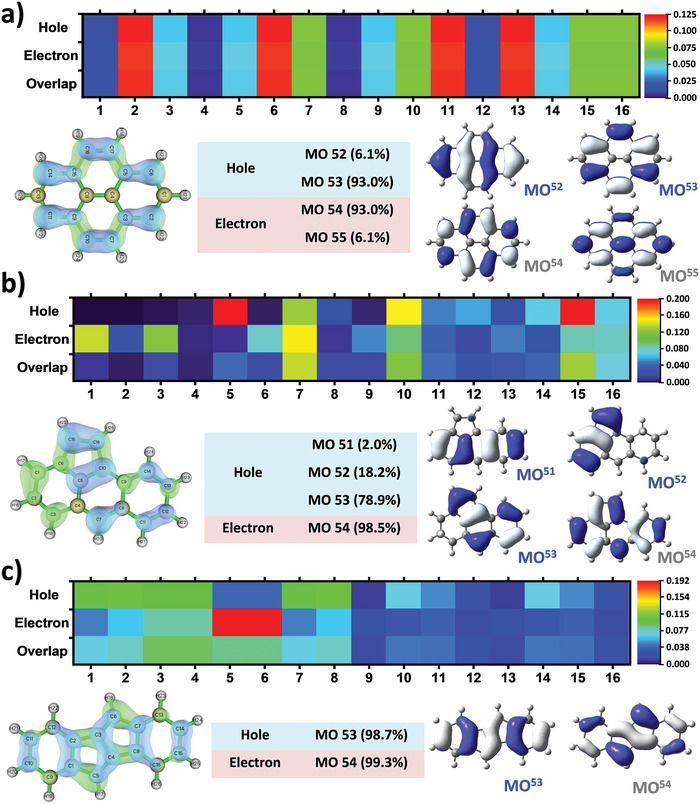
Hole‐electron analysis of *S*_0_→*S*_1_ transition for a) Py, b) AAE and c) DIN, respectively. With the sequence of atomic contributions to hole and electron in terms of heat map, distribution of hole and electron on molecular skeleton at the same time (green represents the electron distribution, and blue represents the hole distribution), MOs with contribution to hole or electron >1%.

To comprehensively investigate the electron excitation characteristics of the pure PAHs, a hole‐electron analysis utilizing the Multiwfn program was conducted.^[^ ^16^ ^]^ It was observed that for the Py core, the distance of charge transfer (*D* index) during the *S*_0_→*S*_1_ excitation was nearly zero, while the *S*r value (integral of *S*r function overlap) approached the upper limit of 1 (Table S3, Supporting Information), indicating a typical local excitation (LE) type transition from *S*_0_ to *S*_1_ in Py. Upon introducing a single five‐membered ring (as in the cases of FLA, APD, and AAE), the *D* indexes significantly increased while the *S*r values decreased, implying the involvement of charge transfer (CT) excitation to varying degrees and contributing to a lower excitation energy for the *S*_0_→*S*_1_ transition. However, due to the highly symmetrical geometry of DCH and DIN, their excitations were tentatively identified as holistic LE characteristics instead. To further visually grasp the fundamental differences, heat maps of charge transfer matrices, focusing on the dominant carbon atoms among the different PAHs, were generated (**Figure** **3**; Figures S9 and S10, Supporting Information). It is noteworthy that the *S*_0_→*S*_1_ excitation in DIN was predominantly localized within the central pentalene moiety, with the hole primarily occupying on MO53 (98.7%, HOMO), while the electron largely residing on MO54 (99.3%, LUMO). In addition, the hole distribution was mainly encompassed on the C1‐C4 and C7‐C8 atoms and constituting the strongly antiaromatic ground state (Figure 2f_1_), whereas the electron was predominantly confined to the C5‐C6 atoms and yielding the highly aromatic excited state (Figures 2f **_2_** and 3c). Consequently, such naturally led to the lowest excitation energy and *E*_S1_ state for DIN.

**Figure 4:**
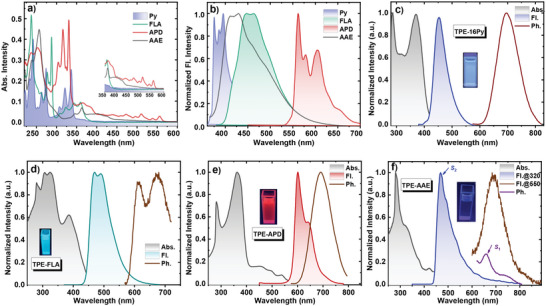
a) UV–vis absorption and b) emission spectra of these pyrene isomers, all measured in a dilute dichloromethane solution (1 × 10^−5^ M). The absorption (Abs.), fluorescence (Fl.) and phosphorescence (Ph.) spectra of c) TPE‐16Py, d) TPE‐FLA, e) TPE‐APD and f) TPE‐AAE (the labels Fl. @320 and Fl. @550 indicate that the excitation wavelengths were 320 and 550 nm, respectively), all obtained from dilute toluene solutions (1 × 10^−5^ M).

With the purpose of achieving full‐color displays and high efficiency for non‐doped pure hydrocarbon emitters, four C_16_H_10_ isomerides (Py, FLA, APD, and AAE) incorporating dual TPE units, namely TPE‐16Py, TPE‐FLA, TPE‐APD, and TPE‐AAE, were synthesized and well characterized (Scheme S1 and Figures S1–S6, Supporting Information). Initially, the UV–vis absorption and photoluminescence (PL) spectra of these core structures were examined in dilute toluene. As depicted in **Figure** **4**a, all of these PAHs exhibited intricate fine π–π* electron excitations within the 300–400 nm range, with the absorption edges of APD and AAE cores notably extending into the 600 nm region. Such difference could be attributed to the existence of weak short‐range CT transitions in the latter two. Obviously, the emission spectra of these cores spanned the entire visible region, ranging from near‐ultraviolet (Py) to deep blue (AAE), blue‐green (FLA), and finally red (APD) (Figure 4b). With the incorporation of neutral TPE rotator, the absorption and emission bands experienced partial degeneracy and exhibited slight red‐shifts toward the visible region (Figure 4c–f). Accordingly led to a gradual decrease in the energy of *E*_S1_, from 2.74 eV (TPE‐16Py) to 2.53 eV (TPE‐FLA), 2.06 eV (TPE‐APD) and finally 1.88 eV (TPE‐AAE). Interestingly, the energy difference among the lowest triplet excited state (*E*_T1_) of the aforementioned four compounds was unobvious (≈1.80 eV). As a consequence, this led to a gradual decrease in the Δ*E*_ST_ values, reaching merely 0.15 and 0.08 eV for TPE‐APD and TPE‐AAE, respectively, presenting an innovative avenue for realizing TADF characteristics in pure hydrocarbon emitters. Unexpectedly, compared to the previous theoretical results, the abnormal PL spectra observed for both AAE and TPE‐AAE (Figure 4b,f) can be attributed to the anti‐Kasha emission feature of the AAE core. Accordingly, vibration analysis and transition rate calculations were performed using the MOMAP program package in the DUSHIN module to examine their luminescence process.^[^ ^17^ ^]^ Evidently, exemplifying with AAE as a case study, the calculated *k*_r_ value for *S*_2_ (2.66 × 10^8^ s^−1^) was twelve times higher than that of *S*_1_ (2.02 × 10^7^ s^−1^), accompanied by a substantial decrease in the *k*_ic_ and reorganization energy (*E*_λ_) of *S*_2_ compared to *S*_1_ (Figure S11, Supporting Information). Moreover, the HRFs of AAE‐cored emitters at both high‐ and low‐frequency vibrational modes were significantly suppressed in *S*_2_ compared to *S*_1_, thus providing a more favorable radiative pathway for the *S*_2_→*S*_0_ transition than *S*_1_→*S*_0_.

**Figure 5:**
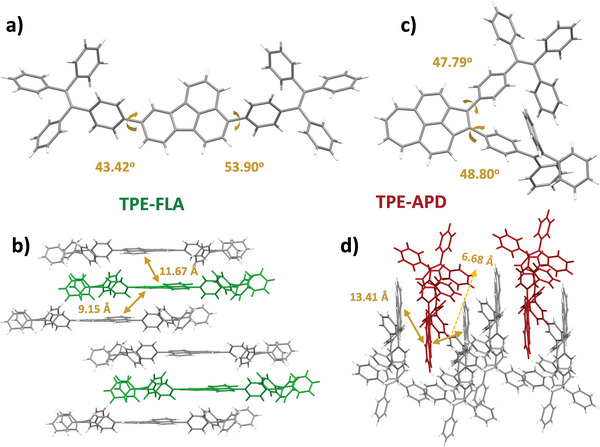
Single‐crystal structure and aggregation stacking model of the a)/b) TPE‐FLA and c)/d) TPE‐APD, respectively (CCDC 2 283 338 and 2 283 340).

The aggregation‐induced emission enhancement (AIEE) characteristic of these pure hydrocarbon emitters was investigated in a mixed solvent of water/tetrahydrofuran or ethanol/toluene.^[^ ^18^ ^]^ Notably, all these emitters exhibited pronounced AIEE properties (Figure S14, Supporting Information). The highest fluorescence intensity for TPE‐APD was observed at a water proportion of ≈60%, while TPE‐FLA and TPE‐16Py achieved the maximum brightness at a high water (or ethanol) proportion of 90%, indicating their suitability as candidates for non‐doped emitting layers. The significant differences can be attributed to their distinguished packing models in the aggregated states. As depicted in **Figure** **5**a,c, both TPE‐APD and TPE‐FLA displayed moderate dihedral angles (≈44° to 54°) between the PAH cores and the peripheral TPE units. Due to the disparity of steric hindrance between the position 3 and 8 of FLA, the dihedral angles between the TPE moieties and FLA core were obvious difference in contrast to the equivalent substitution sites in TPE‐APD. Moreover, the distances between adjacent luminescent cores were differed in the aggregated states as well (Figure 5b,d), and affording distinct AIEE features. For instance, the minimum spacing between two central cores reached as low as 6.68 Å when two TPE units were positioned on one side (TPE‐APD), while this situation was notably enhanced in the case of bilaterally distributed ones (TPE‐FLA). Owing to their AIEE attributes, all these TPE‐modified PAH emitters prominently exhibit heightened photoluminescence quantum yields (PLQY) when incorporated into heavily doped 26DCzPPy matrices (50 wt.%), with the PLQY of TPE‐16Py and TPE‐FLA even approaching 100% (**Table** **1**).

**Table 1:** Photophysical, electrochemical, and transient spectra data of these TPE‐based PAH emitters.

Compounds	λ_abs_ ^a)^ [nm]	λ_em_ ^a)^ [nm]	*E*_g_ ^b)^ [eV]	*E*_S1_ ^c)^ [eV]	Δ*E*_ST_ ^d)^ [eV]	*E*_HOMO_/ *E*_LUMO_ ^e)^ [eV]	PLQY^f)^	τ_p_ [ns]/ τ_d_ [µs]^g)^
TPE‐16Py	287, 317, 371	453	2.95	2.74	0.93	−5.55/ −2.89	95%/ 86%	1.85/ –^h)^
TPE‐FLA	306, 325, 386	491, 469	2.73	2.53	0.71	−5.52/ −2.79	92%/ 84%	3.99/ –^h)^
TPE‐APD	365, 455, 587	602, 641	2.04	2.06	0.15	−5.32/ −3.28	3%/ 2%	1.80/ 8.76
TPE‐AAE	322, 420, 516	468, 659	2.05	1.88	0.08	−5.35/ −3.30	–/ –^h)^	–/ –^h)^

^a)^ The characteristic absorption and emission peaks were obtained in dilute toluene (1 × 10^−5^ M);

^b)^ estimated from the cutting edge of absorption spectra at long‐wavelength direction;

^c)^ calculated from the dominant emission peaks;

^d)^ deduced from the onset of fluorescence and phosphorescence spectra at 77 K;

^e)^ HOMOs is evaluated from the oxidation process of cyclic voltammetry curves with reference to the internal standard of ferrocene, LUMOs is calculated by *E*_HOMO_s and *E*_g_s;

^f)^ measured in films (50 wt.% doped in 26DCzPPy matrix)/ toluene (1 × 10^−5^ M);

^g)^ fitting from the transient spectra of doped films, and;

^h)^ nonexistence or no valid data can be obtained.

**Figure 6:**
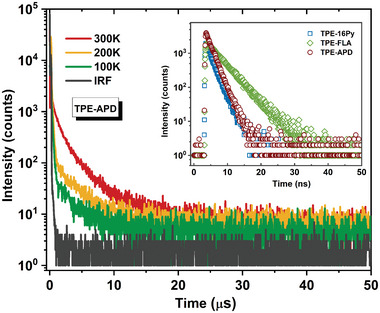
Temperature‐dependent transient photoluminescence spectra of the TPE‐APD film (50 wt.% doped in 26DCzPPy matrix), inset: transient photoluminescence spectra of the TPE‐16Py, TPE‐FLA, and TPE‐APD (50 wt.% doped in 26DCzPPy matrix) in short scale.

Considering the calculated *T*_4_ levels in both TPE‐16Py and TPE‐FLA are only slightly higher than the *S*_1_, as well as the spin‐orbit coupling (SOC) values between the aforementioned two orbitals are significantly larger than the others (Figure S7, Supporting Information), which raise the prospect of a potential hybridized local and charge‐transfer (HLCT) mechanism existing within these two entities. In order to verify this hypothesis, a series of absorption and fluorescence spectra were compiled in diverse solvent environments (Figure S15, Supporting Information). Regrettably, due to the limited solubility of TPE‐16Py, comprehensive spectra were not obtainable across multiple solvents. Despite the dominant transition in pure hydrocarbon emitters is of the LE type, unconspicuous CT characteristics in both TPE‐FLA and TPE‐APD could be distinguished from their absorption spectra (Figure 4; Figure S15, Supporting Information). By analyzing the abrupt change in the slope of the Stokes shifts (*ν*_a–_ *ν*_f_) with respect to solvent polarities (*f*), we inferred the presence of a HLCT feature for TPE‐FLA (Figure S16, Supporting Information), which is of significant i As a consequence, importance for facilitating the harvesting of typically inaccessible triplet excitons in OLEDs.^[^ ^19^ ^]^ Furthermore, fluorescence lifetimes were ascertained via transient photoluminescence decay measurements, unveiling swift prompt fluorescence decay in heavily doped films (50 wt.%). Fitted lifetimes of 1.85, 3.99, and 1.80 ns were determined for TPE‐16Py, TPE‐FLA, and TPE‐APD, respectively (**Figure** **6**). Notably, TPE‐APD, both in dilute toluene solution and heavily doped thin films, exhibited characteristic second‐order exponential PL decay, as depicted in Figure 6 and Figure S17 (Supporting Information). Additionally, the delay ratio of TPE‐APD showed a marked decrease with the reduction of temperature, confirming the uncommon occurrence of TADF behavior in pure hydrocarbon emitters. The fitted lifetime for the delayed components (*τ*_d_) of the doped sample was determined to be 8.76 µs at 300 K, which aligns with the relatively small energy gap (Δ*E*_ST_) of 0.15 eV and a considerable SOC value of 1.101 cm^−1^ between the *S*_1_ and *T*_1_ states.

**Figure 7:**
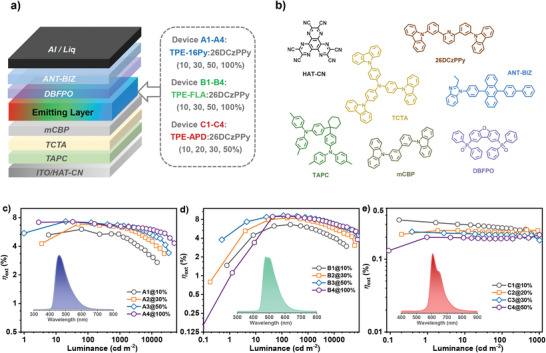
a) Schematic diagram of the device structures with variable emitting layers, and b) chemical structures of the auxiliary materials involved in the devices. The *η*_ext_ versus luminance of the c) TPE‐16Py, d) TPE‐FLA, and e) TPE‐APD based devices (insert: electroluminescence spectra at 5 V).

Electroluminescent (EL) devices with the configuration of “ITO/dipyrazino[2,3‐*f*:2′,3′‐*h*]quinoxaline‐2,3,6,7,10,11‐hexacarbonitrile (HAT‐CN, 5 nm)/1,1‐bis((di‐4‐tolylamino)phenyl)‐cyclohexane (TAPC, 30 nm)/4,4′,4′'‐tris(carbazol‐9‐yl)triphenyl‐amine (TCTA, 15 nm)/3,3‐di(9*H*‐carbazol‐9‐yl)biphenyl (mCBP, 10 nm)/PAH emitters:26DCzPPy (*x*%, *x* = 10, 20, 30, 50 or 100, 30 nm)/dibenzo[*b*,*d*]furan‐2,8‐diylbis(diphenylphosphine oxide) (DBFPO, 20 nm)/1‐(4‐(10‐([1,1′‐biphenyl]−4‐yl)anthracen‐9‐yl)phenyl)−2‐ethyl‐1H‐benzo[*d*]imidazole (ANT‐BIZ, 30 nm)/Liq (2 nm)/Al”, were fabricated to assess the suitability of these PAH emitters in OLED. The emitting layers, with different doping ratios of TPE‐16Py, TPE‐FLA, or TPE‐APD, were denoted as device A1‐4, B1‐4, or C1‐4, respectively (**Figure** **7**a). Interestingly, even without using any sensitizing host materials, the *η*_ext,max_ of TPE‐16Py and TPE‐FLA‐based fluorescent devices all exceeded the theoretical threshold of 5% for conventional fluorescence‐based OLEDs (Figure 7c,d; Figure S18, Supporting Information). This can be attributed to the very high horizontal orientation factors (Θ//≈100%, Figure S19, Supporting Information), high PLQY as well as the possible contribution of HLCT mechanism (Figure S16, Supporting Information). In contrast, the inferior EL performance of devices C1‐C4 may be primarily attributed to the low PLQY of TPE‐APD (Table 1), although the observation of a narrow Δ*E*_ST_ (0.15 eV) and short delayed fluorescence lifetime (*τ*_d_ = 8.76 µs) implied a high exciton utilization. Nevertheless, the full‐color EL emission can be preliminarily realized on the three pure hydrocarbon isomerides without incorporating any heteroatoms or expanding the π‐delocalization.

Notably, with the doping ratio raised to 100%, negligible degradation of EL performances was achieved for both A4 and B4, with the *η*_c,max_/*η*_p,max_/*η*_ext,max_ of 15.1 cd A^−1^/12.9 lm W^−1^/7.2% and 22.9 cd A^−1^/17.7 lm W^−1^/9.1% for TPE‐16Py and TPE‐FLA based devices (**Table** **2**), respectively. Moreover, both A4 and B4 shared a comparable low‐efficiency roll‐offs at 1000 cd m^−2^ (9.7% for A4 and 7.7% for B4) outperforming all the doped cases, which deliver the best results among non‐doped pure hydrocarbon‐based emitting layers to date.^[^ ^1^, ^7^ ^]^ Interestingly, in non‐doped devices, the turn‐on voltages (*V*_on_) showed varying degrees of reduction compared to the doped devices, indicating favorable and balanced charge injection and transport in pure PAHs. These findings highlight the potential of pure hydrocarbon emitters for further advancements in EL performance.

**Table 2:** Key data of these pure hydrocarbon emitter‐based devices.

Devices	*V*_on_ ^a)^ [V]	*L*_max_ ^b)^ [cd m^−2^]	*η*_c,max_/*η*_p,max_ ^c)^ [cd A^−1^]/ [lm W]	*η*_ext_ ^d)^ [%]	CIE^e)^ [x, y]
A3	4.0	33 733	13.2/9.4	7.3/6.6	[0.18, 0.23]
A4	3.5	50 230	15.1/12.9	7.2/6.5	[0.22, 0.28]
B3	3.3	75 461	23.3/17.3	9.2/8.5	[0.22, 0.42]
B4	3.1	77 731	22.9/17.7	9.1/8.4	[0.22, 0.43]
C1	4.3	848	0.47/0.36	0.35/–	[0.60, 0.35]

^a)^ Turn‐on voltage refer to the brightness of 1 cd m^−2^;

^b)^ the maximum brightness;

^c)^ the maximum current efficiency and the maximum power efficiency of the device;

^d)^ the external quantum efficiency of the devices, following the sequence of maximum value, and at 1000 cd m^−2^, respectively;

^e)^ the CIE coordinate of the devices.

## 3 Conclusion

In conclusion, we demonstrated a versatile strategy for significantly modifying the fluorescence characteristics of pure hydrocarbon‐based emitters, without the incorporation of heteroatoms or alterations to their π‐extension. Notably, among the different pyrene isomerides, the introduction of antiaromatic five/seven‐membered rings leads to drastic changes in emission peaks, ranging from ultraviolet to near‐infrared regions, along with the observation of delayed fluorescence and anti‐Kasha emission. The abnormal emission properties were extensively investigated through theoretical calculations and photophysical studies, revealing the critical role of aromaticity/anti‐aromaticity intensity and distribution in both ground and excited states. To the best of our knowledge, this work showcases the first example of pure hydrocarbon‐based TADF‐OLEDs, exemplified by TPE‐APD with Δ*E*_ST_ = 0.15 eV and *τ*_d_ = 8.76 µs. Furthermore, both TPE‐16Py and TPE‐FLA exhibit exceptionally high PLQY and Θ//value approaching 100%, making them highly desirable for ideal organic emitters. As a demonstration, when employing them as emitting layers in OLEDs, blue, green, and red devices achieve *η*_ext,max_ of 7.2%, 9.2%, and 0.35%, respectively. Of particular note that the TPE‐FLA‐based device achieves an *η*_ext,max_ of 9.1% with a minimal efficiency roll‐off (7.7% at 1 000 cd m^−2^), thus yielding the best performance among non‐doped pure hydrocarbon‐based devices. This work not only provides deep insights into understanding the luminous mechanism of pure hydrocarbon‐based emitters but also introduces a novel concept for designing efficient organic emitters beyond pure hydrocarbons.

## Conflict of Interest

The authors declare no conflict of interest.

## Supporting information

Supporting Information

## Data Availability

The data that support the findings of this study are available from the corresponding author upon reasonable request.
