# Supplementary material for: Pure Polycyclic Aromatic Hydrocarbon Isomerides with Delayed Fluorescence and Anti‐Kasha Emission: High‐Efficiency Non‐Doped Fluorescence OLEDs
Source: Adv Sci (Weinh). 2023 Sep 17;10(31):2304204. doi: 10.1002/advs.202304204 (PMC10625133; doi:10.1002/advs.202304204)
Supplement: Supplementary file 1 — Supporting Information [file ADVS-10-2304204-s001.pdf]

ADVANCED SCIENCE
Open Access

Supporting Information

for *Adv. Sci.*, DOI 10.1002/advs.202304204

Pure Polycyclic Aromatic Hydrocarbon Isomerides with Delayed Fluorescence and Anti-Kasha Emission: High-Efficiency Non-Doped Fluorescence OLEDs

Haoxin Huang, Nengquan Li, Shuguang Fu, Xuechao Mo, Xiaosong Cao, Xiaojun Yin and Chuluo Yang*

Supporting Information

Pure Polycyclic Aromatic Hydrocarbon Isomerides with Delayed Fluorescence and Anti-Kasha Emission: High-Efficiency Non-Doped Fluorescence OLEDs

Haoxin Huang, Nengquan Li, Shuguang Fu, Xuechao Mo, Xiaosong Cao, Xiaojun Yin & Chuluo Yang*

H. Huang, Dr. N. Li, S. Fu, X. Mo, Dr. X. Cao, Dr. X. Yin & Prof. C. Yang
Shenzhen Key Laboratory of New Information Display and Storage Materials, College of Materials Science and Engineering, Shenzhen University, Shenzhen, 518060 (P. R. China).
E-mail: xiaojunyin@szu.edu.cn, clyang@szu.edu.cn

Table of Contents

General Informations 2
Theoretical Calculations 2
Device Fabrication and Characterization 3
Materials and Methods 3
Synthesis and Characterization 4
Theoretical calculations 11
Thermal properties 16
Electrochemical property 16
Photophysical properties 17
Application in organic electroluminescent devices 19
References 20

General Informations

^{1}H and ^{13}C nuclear magnetic resonance (NMR) spectra were recorded on Bruker AV 500M NMR spectrometer at room temperature, using $CDCl_3$ or CD_2Cl_2 as solvent with tetramethylsilane as internal standard. High resolution mass spectrometer (HRMS) were collected on a Thermo Scientific LTQ Orbitrap XL mass spectrometer with electron spray ionization. Thermogravimetric analysis (TGA) was recorded on a TA Q50 instrument under nitrogen atmosphere with an identical heating rate of 10 ^{o}C per min from r.t. to 650 ^{o}C. The degradation temperature (T_d) was estimated from the 5% weight loss. Cyclic voltammetry (CV) measurements were carried out on a CHI600 electrochemical analyzer (Chenhua, China) at room temperature, with a conventional three-electrode system consisting of a glassy carbon working electrode, a platinum wire auxiliary electrode, and an Ag/AgCl standard electrode was used as the reference electrode. The supporting electrolyte was 0.1 M tetrabutylammonium hexafluorophosphate (n-Bu_4NPF_6) in anhydrous dichloromethane solution, and ferrocene was additional estimated as the internal standard during the measurement. UV-Vis spectra in solution were recorded on a UV-3100 spectrophotometer at room temperature. Room-temperature photoluminescence spectra and phosphorescence spectra were measured on a Hitachi F-7000 fluorescence spectrophotometer with xenon lamp as the light source. The absolute fluorescence quantum yields (PLQY) were measured on a Quantaurus-QY measurement system (C9920-02, Hamamatsu Photonics) equipped with a calibrated integrating sphere. During the PLQY measurements, the integrating sphere was purged with pure and dry argon to maintain an inert environment. The lifetimes of fluorescence and delayed fluorescence were performed on PicoQuant Fluotime300.

Theoretical Calculations

Density functional theory (DFT) calculations of the geometrical and electronic properties of these pure PAHs and TPE-based emitters at ground-states were performed by using Gaussian 16 software package at the B3LYP/6-311G** level including Grimme's dispersion correction.[1] The configuration optimization of the excited states of these PAHs were carried out at the same level. The nucleus-independent chemical shifts (NICS) were calculated using the gauge independent atomic orbital (GIAO) standard at B972/def2TZVP level with the consideration of SMD solvent model in chloroform. To remedy the rapidly damping of non-Coulomb part of exchange functionals at large distances, the long-range corrected functional LC-ωPBE was adopted to calculate the electron excitation energies of the TPE-modified PAHs accurately. An iteration procedure was employed to non-empirically tune the ω parameters

under the optimized geometrical configuration of ground states,[2] and then the time-dependent DFT (TD-DFT) calculations were performed at the LC-ωPBE/6-311G(d,p) level of theory with tuned ω values. All calculations were performed in the gas phase, and visualized using GaussView 6.0. The wave function analysis, hole-electron analysis and visualization of two-dimensional iso-chemical shielding surface (2D-ICSS) was carried out by using Multiwfn 3.8 program.[3] The theoretical spectral simulation, vibration analysis and all kinds of transition rates were simulated by using MOMAP (Molecular Materials Property Prediction Package) in the DUSHIN module.[4-7]

Device Fabrication and Characterization

The ITO coated glass substrates [ZhongNuo Advanced Material (Beijing) Technology Co., Ltd] with a sheet resistance of 15 Ω square^{-1} were consecutively ultrasonicated with acetone/isopropanol and dried with nitrogen gas flow, followed by 20 min ultraviolet lightozone (UVO) treatment in a UV-ozone surface processor (PL16 series, Sen Lights Corporation). Afterwards, the ITO substrates was transferred to the deposition system. The organic layers including dipyrazino[2,3-*f*:2′,3′-*h*]quinoxaline-2,3,6,7,10,11-hexacarbonitrile layer, 1,1-bis((di-4-tolylamino)phenyl)-cyclohexane layer, 4,4′,4′′-tris(carbazol-9-yl)triphenyl-amine layer, 3,3-di(9Hcarbazol-9-yl)biphenyl layer, emitting layer, dibenzo[*b*,*d*]furan-2,8-diylbis(diphenylphosphine oxide layer, 1-(4-(10-([1,1′-biphenyl]-4-yl)anthracen-9-yl)phenyl)-2-ethyl-1*H*-benzo[*d*]imidazole layer, Liq layer were successively deposited by thermal evaporation at 5×10^{-5} Pa with rates of 0.2-3 Å/s. The cathode Al layer were deposited with rates of 3 Å/s. The emitting area of the device is 0.09 cm^2. The current density-voltage-luminance (*J*-*V*-*L*) properties and external quantum efficiency curves and electroluminescence spectra were measured using a Keithley 2400 source meter and an absolute EQE measurement system (C9920-12, Hamamatsu Photonics, Japan).

Materials and Methods

All the commercially available reagents and solvents used in this work were used directly unless otherwise mentioned. Key intermediates of 1,6-dibromopyrene (16Py-2Br) was purchased from Shanghai Haohong Biomedicine Technology Co. LTD, and the rest three 3,8-dibromofluoranthene (FLA-2Br), 1,2-dibromocyclohepta[*fg*]acenaphthylene (APD-2Br) and aceanthrylen-2-yltrimethylsilane (AAE') were synthesized according to the previous literatures.[8,9]

Synthesis and Characterization

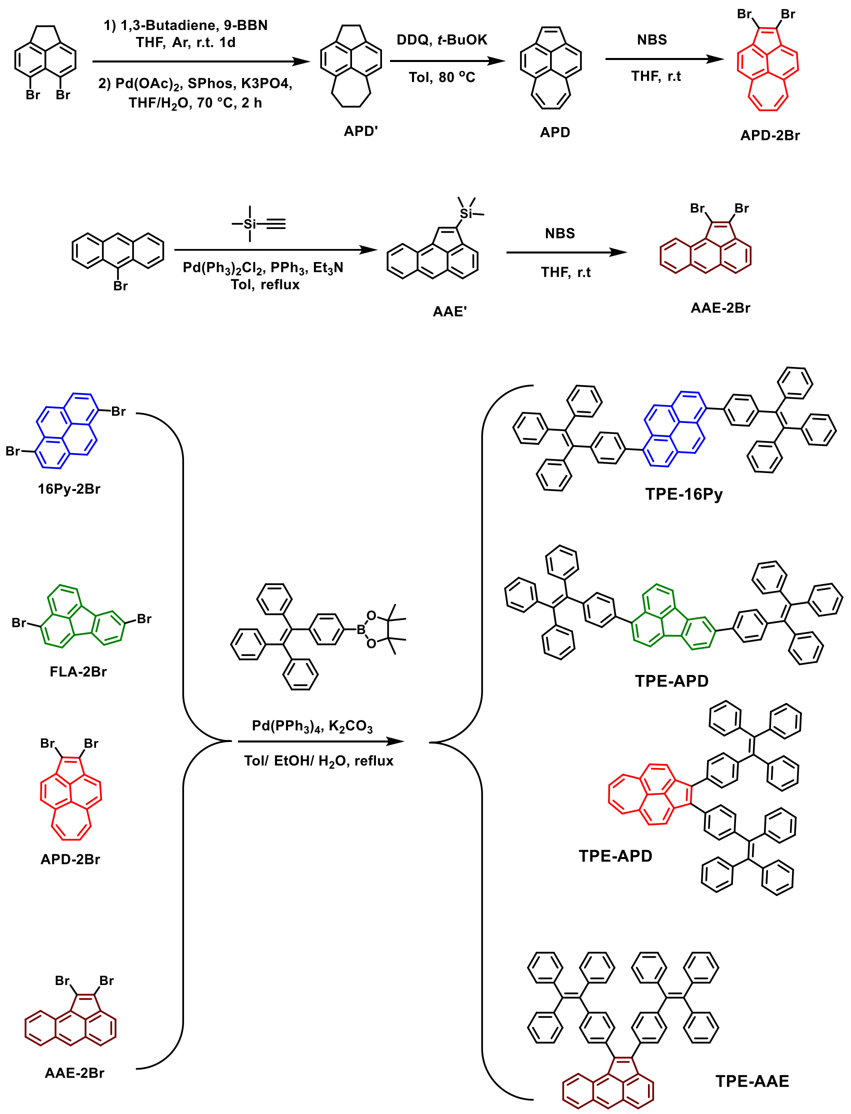

Scheme S1. Synthesis routes of these pure polycyclic aromatic hydrocarbon intermediates and the corresponding TPE modified emitters.

The synthetic route of APD-2Br was executed according to the previously reported literature[8] with appropriate modification. Initially, the starting materials of 1,3-butdiene (19.2 mL, 38.4 mmol, 2.0 M in THF) and 9-BBN (154 mL, 76.8 mmol, 0.5 M in THF) were added to a round bottom flask under N_2 atmosphere. The mixture, which have reacted at room temperature for 1 day, was delivered to another round bottom flask charged with $Pd(OAc)_2$ (717 mg, 3.20 mmol), SPhos (2.63 g, 6.40 mmol), K_3PO_4 (20.40 g, 96.00 mmol) and 3,4-dibromoacenaphthene (10.00 g, 32.00 mmol) under argon. Additional THF (270 mL) and H_2O (50 mL) was injected into the flask, the combined mixture was stirred at 70 °C for another 1

day. After cooling to room temperature, the mixture was extracted with EtOAc for three times. The combined organic layers were washed with water and brine and dried over $MgSO_4$. Removing the solvent under reduced pressure, the crude product was chromatographically purified on silca gel column. The eluent is pure petroleum. White solid 1,2,5,6,7,8-hexahydrocyclohepta[*fg*]acenaphthylene (APD') was abtain by further recrystallization from DCM/MeOH via diffusion with good yield of 85%. The obtained white solid (2.80 g, 13.4 mmol), DDQ (18.34 g, 80.60 mmol), *t*-BuOK (15.08 g, 134.5 mmol) and toluene (260 mL, 0.05 M) was then added to a round bottom flask successively. The combined mixture was heated to 80 ^{o}C and stirring for 17 h. After cooling down, the suspension was filtered via diatomite, and the filtrate was heated under reduced pressure to remove the redundant solvent. The crude product was chromatographically purified on silca gel column with petroleum as eluant to give red solid cyclohepta[*fg*]acenaphthylene (APD) at a good yield of 82%. Afterwards, APD (600 mg, 2.97 mmol) was dissolved in THF (600 mL, 5 mM) in a round bottom flask. After adding NBS (1.12 g, 6.24 mmol) portionwise, the combined mixture was stirred at room temperature for 12 h. After the reaction finished, quenched it with H_2O. The reacted mixture was extracted with DCM for three times. The organic layers were washed with water and brine, dried over with MgSO4. After removing the solvent under reduced pressure, the crude product was then chromatographically purified on silca gel column, using petroleum ether/DCM (3/1, *v*/*v*). Further purification via recrystallization from DCM/MeOH to obtain the title compound APD-2Br at a considerable yield of 66%.

Synthesis of 3,8-dibromofluoranthene (FLA-2Br). Br_2 (335 mg, 2.10 mmol) was dissolved in 40 mL DCM in a dropping funnel. To a round bottom flask charged with fluoranthene (2.02 g, 1 mmol) and 60 mL DCM, the Br_2 dilute solution was then dropped slowly into the aforementioned mixture. The suspension was stirred at room temperature over night. After quenching with $Na_2S_2O_3$, the combined mixture was filterd, and the residue was wash with EtOH, DCM successively affording faint yellow solid with a desirable yeild of 85%. ^{1}H NMR (500 MHz, $CDCl_3$) δ [ppm]: 8.05 (d, J = 8.5 Hz, 1H), 8.01 (d, J = 1.5 Hz, 1H), 7.94 (d, J = 7.0 Hz, 1H), 7.85 (d, J = 7.5 Hz, 1H), 7.75-7.71 (m, 3H), 7.51 (dd, J = 8.0, 2.0 Hz, 1H). ^{13}C NMR (126 MHz, $CDCl_3$) δ [ppm]: 140.89, 137.45, 136.04, 135.74, 133.49, 131.33, 130.58, 129.93, 129.17, 126.73, 124.99, 122.76, 122.29, 121.76, 121.40, 121.05. HRMS (ESI) m/z calcd for $C_{16}H_8Br_2$ 359.8972, found 359.8968.

Synthesis of 1,2-dibromoaceanthrylene (AAE-2Br). Aceanthrylen-2-yltrimethylsilane (1.38 g, 5.0 mmol) was dissolved in 300 mL THF in a round bottom flask. NBS (2.24 g, 12.5 mmol)

was then added into the solution slowly, the combined mixture was stirred at room temperature for 12 h. After the complete reaction, quenched it with H_2O and extracted with DCM for three times (50 mL * 3). The collected organic layers was washed with water and brine, dried over with $MgSO_4$. After removing the solvent under reduced pressure, the crude product was chromatographically purified on silca gel column, using n-hexane as eluent. Further purification was carried out via recrystal from DCM/MeOH to obtain the title compound as red solid (yield 25%). ^1H NMR (500 MHz, CD_3Cl) δ 9.20 (d, J = 8.8 Hz, 1H), 8.52 (s, 1H), 8.09 (d, J = 8.6 Hz, 1H), 8.03 (d, J = 8.4 Hz, 1H), 7.81 (d, J = 6.7 Hz, 1H), 7.71-7.65 (m, 1H), 7.61 (dd, J = 8.4, 6.8 Hz, 1H), 7.52-7.46 (m, 1H). HRMS (ESI) m/z calcd for $C_{16}H_8Br_2$ 359.8972, found 359.8973.

General procedure for the Pd-catalyzed Suzuki cross-coupling

To a 150 mL round bottom flask, polycyclic aromatic hydrocarbon dibromide precursors (180 mg, 0.05mmol), 4,4,5,5-tetramethyl-2-(4-(1,2,2-triphenylvinyl)phenyl)-1,3,2-dioxaborolane (505mg, 0.11 mmol), $Pd(PPh_3)$ (29 mg, 5%) and K_2CO_3 (691 mg, 0.25 mmol) were added successively. Subsequently, 40 mL toluene, 10 mL water and 10 mL of ethanol was added via syringe, and the mixted suspension was deoxygenated via sustained bubbling with argon. The mixture was allowed to stir at 110 ^oC for 16 hours. After cooling down, the reaction mixture was poured into 300 mL water and extracted with dichloromethane for three times. The collected organic layers is washed with brine and dried over with sodium sulfate. After the solvent was removed under reduced pressure, the crude product was purified by using column chromatography with silica gel as immobile phase and dichloromethane/petroleum ether (v/v = 1/3) as eluent. Further purification was executed by using recrystallization in dichloromethane/methanol mixture, and followed by vacuum sublimation to afford the target pure hydrocarbon emitters with high purity.

1,6-bis(4-(1,2,2-triphenylvinyl)phenyl)pyrene **(TPE-16Py)**: creamy white solid (yield 82%). The purification of **TPE-16Py** was executed with following procedure: after colling down, the obtained suspension was treated with direct filtration, and the residues was washing with a mass of acetone, hot dichloromethane, chloroform, toluene, respectively, and then followed with twice vacuum sublimation. Due to the poor solubility of **TPE-16Py**, no valid NMR data can be collected. HRMS (ESI) m/z calcd for $C_{68}H_{47}^+$ $(M+H)^+$ 863.36723, found 863.36676.

3,8-bis(4-(1,2,2-triphenylvinyl)phenyl)fluoranthene **(TPE-FLA)**: light green solid (yield 88%). ^1H NMR (500 MHz, CD_3Cl) δ [ppm]: 8.11 (s, 1H), 7.96 (dd, J = 10.7, 7.0 Hz, 2H), 7.92 (d, J = 7.9 Hz, 1H), 7.87 (d, J = 8.4 Hz, 1H), 7.62 (dd, J = 8.3, 7.0 Hz, 1H), 7.59 (d, J = 7.3 Hz,

2H), 7.49 (d, *J* = 8.3 Hz, 2H), 7.35 (d, *J* = 8.2 Hz, 2H), 7.20-7.04 (m, 34H). ^{13}C NMR (126 MHz, $CDCl_3$) δ [ppm]: 143.77, 143.67, 142.90, 141.41, 140.62, 140.05, 138.05, 137.01, 135.93, 133.20, 131.87, 131.47, 131.38, 129.57, 128.59, 128.00, 127.82, 127.76, 127.72, 127.68, 127.66, 126.51, 126.23, 121.58, 120.07. HRMS (ESI) m/z calcd for $C_{68}H_{47}^+$ $(M+H)^+$ 863.36723, found 863.36707.

1,2-bis(4-(1,2,2-triphenylvinyl)phenyl)cyclohepta[fg]acenaphthylene **(TPE-APD)**: red powder (yield 42%). ^{1}H NMR (500 MHz, CD_2Cl_2) δ [ppm]: 8.25 (d, *J* = 7.5 Hz, 2H), 7.89 (d, *J* = 7.6 Hz, 2H), 7.67 (dd, *J* = 8.8, 3.7 Hz, 2H), 7.17 (d, *J* = 8.1 Hz, 4H), 7.13-6.96 (m, 34H), 6.84 (dd, *J* = 8.8, 3.6 Hz, 2H). ^{13}C NMR (126 MHz, $CDCl_3$) δ [ppm]: 143.89, 143.77, 143.59, 142.23, 141.27, 140.99, 138.05, 136.87, 135.55, 134.20, 133.75, 131.49, 131.40, 130.24, 127.79, 127.72, 127.67, 127.51, 127.30, 126.97, 126.59, 126.57, 126.49, 125.27. HRMS (ESI) m/z calcd for $C_{68}H_{47}^+$ $(M+H)^+$ 863.36723, found 863.36700.

1,2-bis(4-(1,2,2-triphenylvinyl)phenyl)aceanthrylene **(TPE-AAE)**: dark red powder (yield 30%). ^{1}H NMR (500 MHz, CD_2Cl_2) δ [ppm]: 8.43 (s, 1H), 8.02 (d, *J* = 9.0 Hz, 1H), 7.96 (d, *J* = 8.5 Hz, 1H), 7.74 (d, *J* = 7.5 Hz, 2H), 7.55 (t, *J* = 8.0 Hz, 1H), 7.34 (t, *J* = 7.0 Hz, 1H), 7.27 (t, *J* = 8.0 Hz, 1H), 7.13-6.86 (m, 38H). Due to the poor solubility, no valid ^{13}C NMR can be obtained. HRMS (ESI) m/z calcd for $C_{68}H_{47}^+$ $(M+H)^+$ 863.36723, found 863.36749.

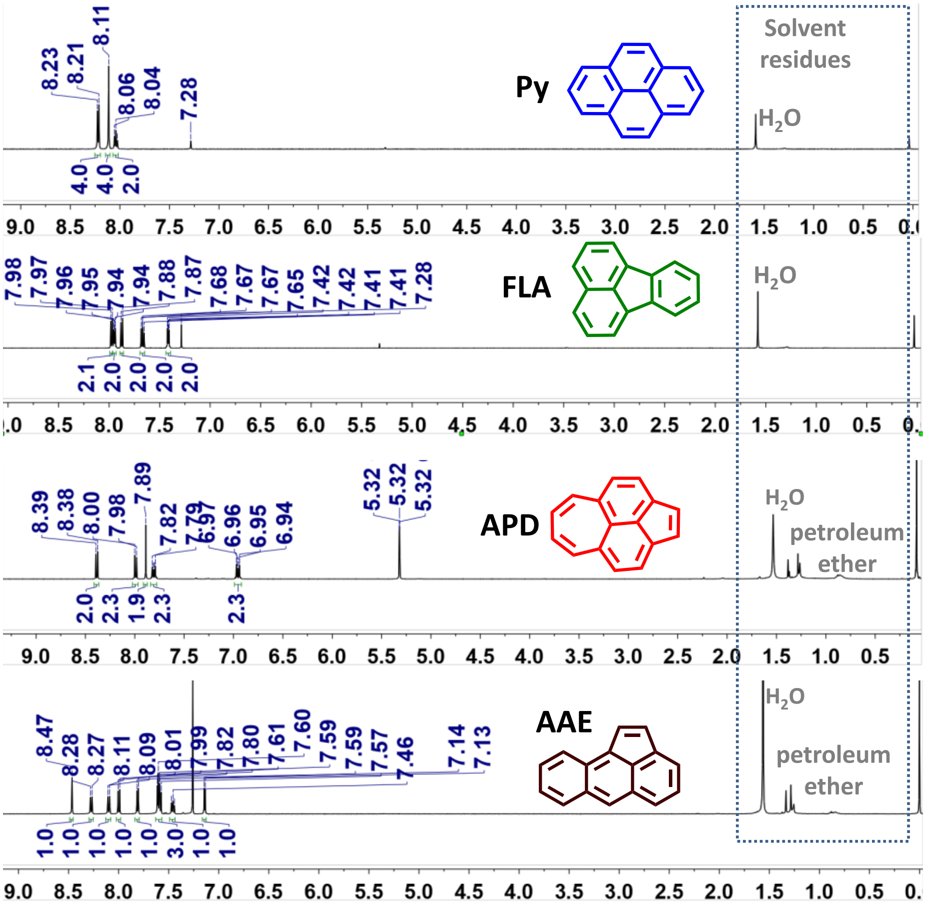

Figure S1. Comparative diagram of the ^{1}H NMR spectra among Py, FLA, APD, and AAE.

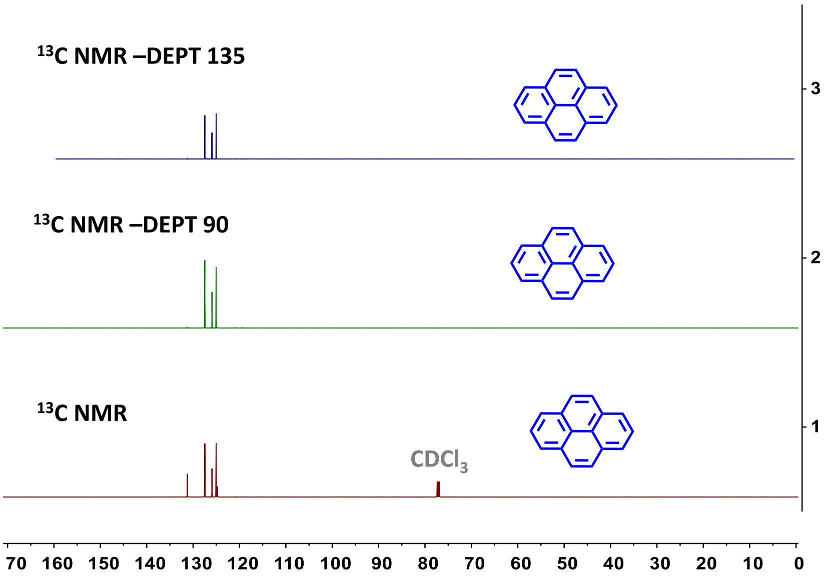

Figure S2. Distortionless enhancement by polarization transfer (DEPT) analysis of the Py.

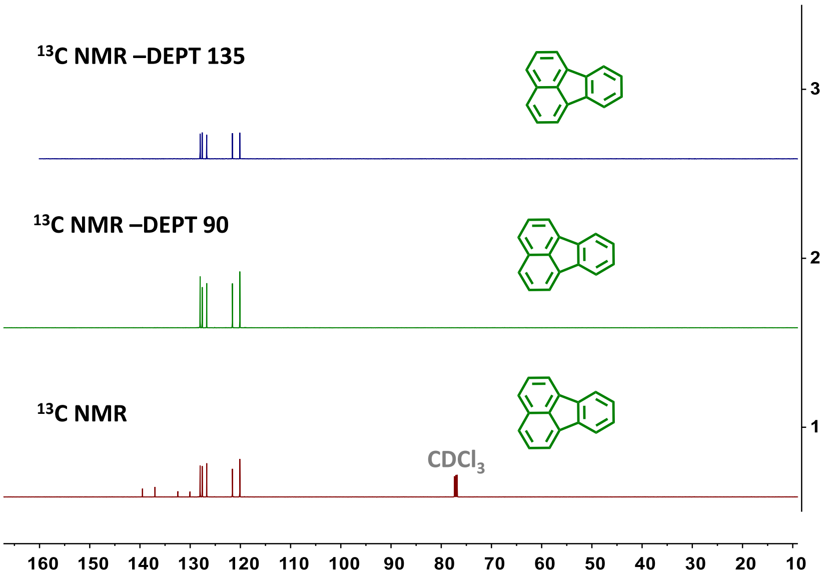

Figure S3. DEPT analysis of the FLA.

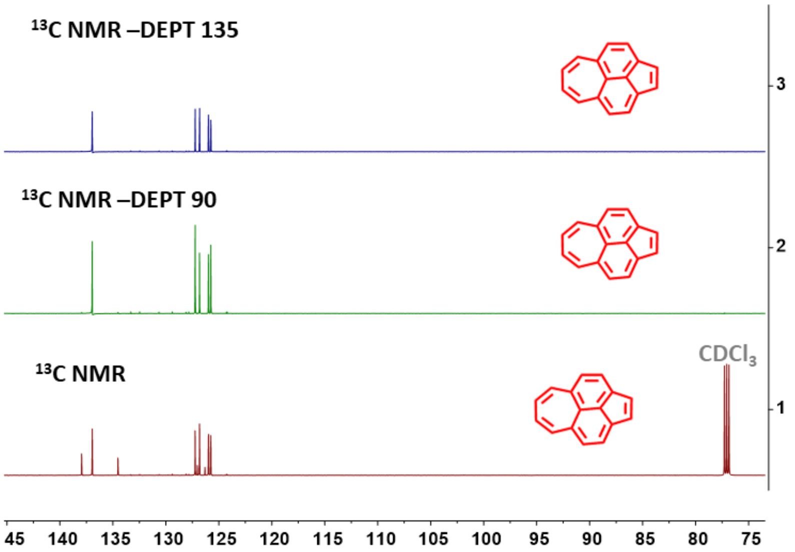

Figure S4. DEPT analysis of the APD.

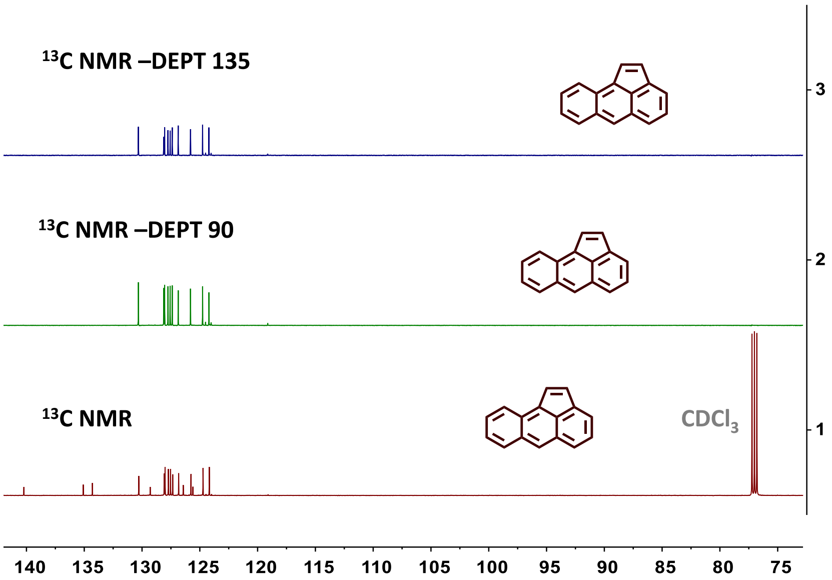

Figure S5. DEPT analysis of the AAE.

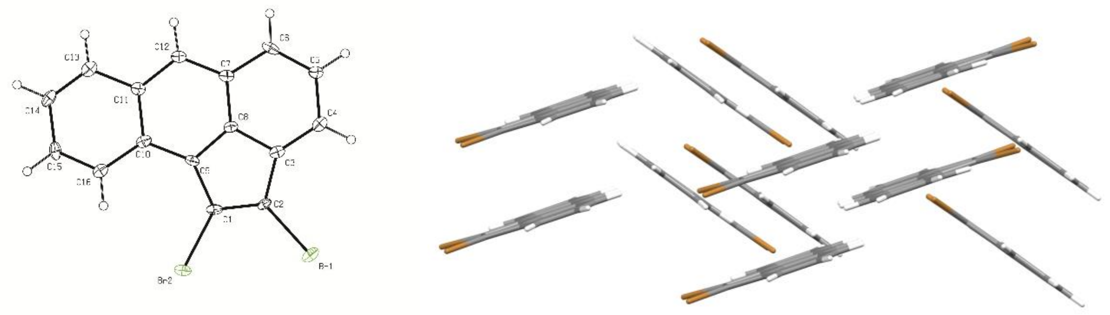

Figure S6. Single-crystal structure of the key intermediate AAE-2Br (left) and corresponding packing model (right), with CCDC deposition number 2283339.

Theoretical calculations

Table S1. Visualized molecular orbitals of these $C_{16}H_{10}$ isomerides, with isovalue of 0.03.

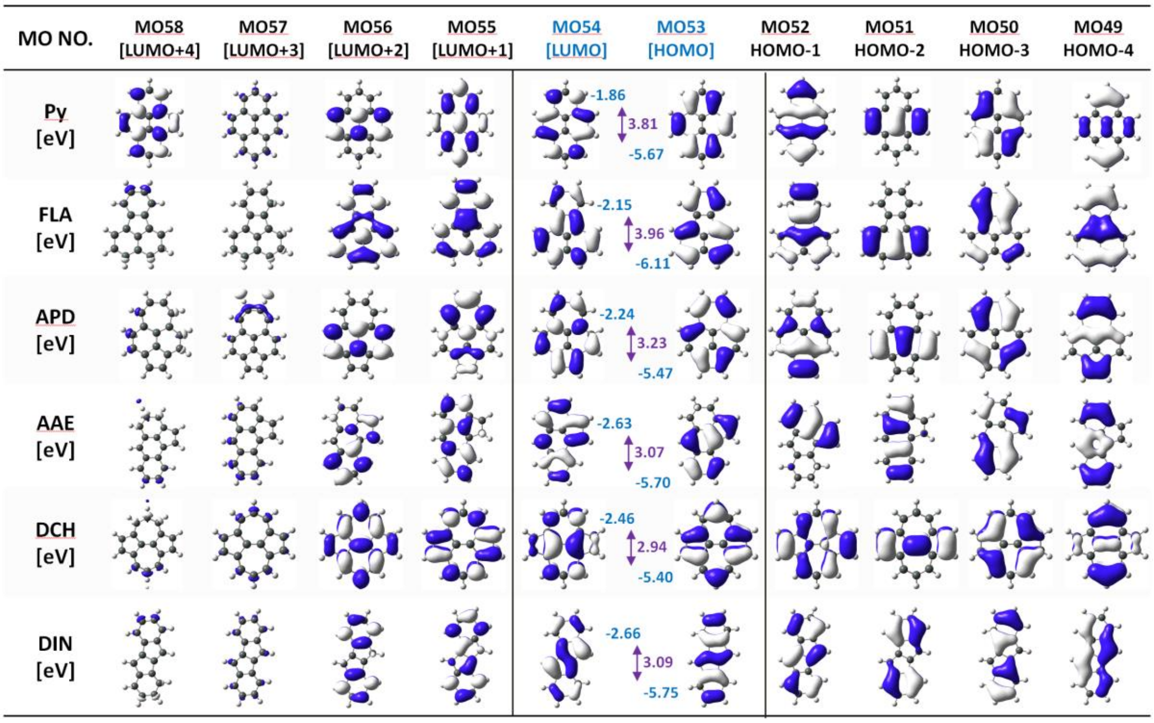

Table S2. Visualized molecular orbitals of the TPE-based pure PAH isomerides, with isovalue of 0.02.

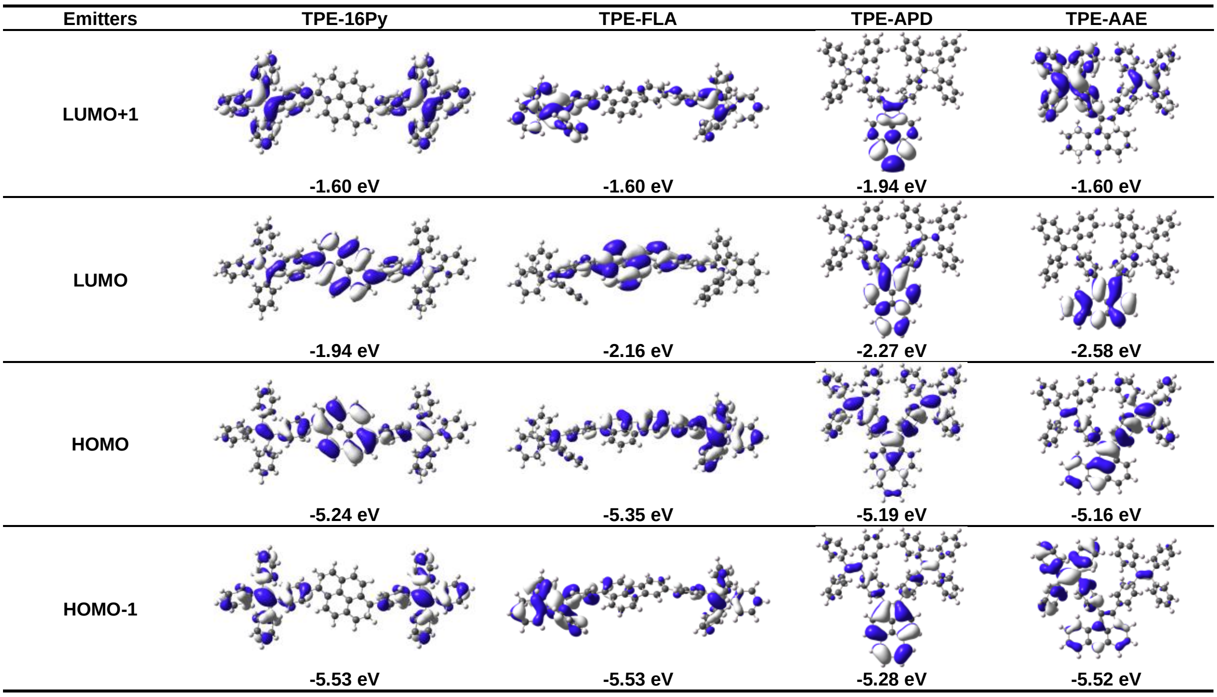

Emitters	TPE-16Py	TPE-FLA	TPE-APD	TPE-AAE
LUMO+1	-1.60 eV	-1.60 eV	-1.94 eV	-1.60 eV
LUMO	-1.94 eV	-2.16 eV	-2.27 eV	-2.58 eV
HOMO	-5.24 eV	-5.35 eV	-5.19 eV	-5.16 eV
HOMO-1	-5.53 eV	-5.53 eV	-5.28 eV	-5.52 eV

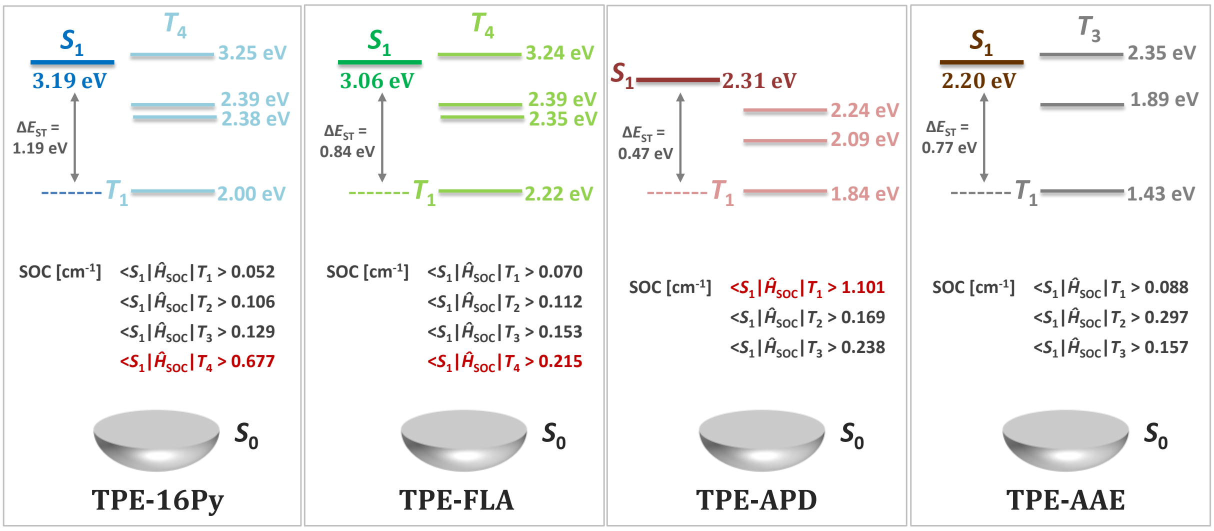

Figure S7. Calculated excited state energy levels and spin-orbit coupling (SOC) values of the four TPE-modified PAHs.

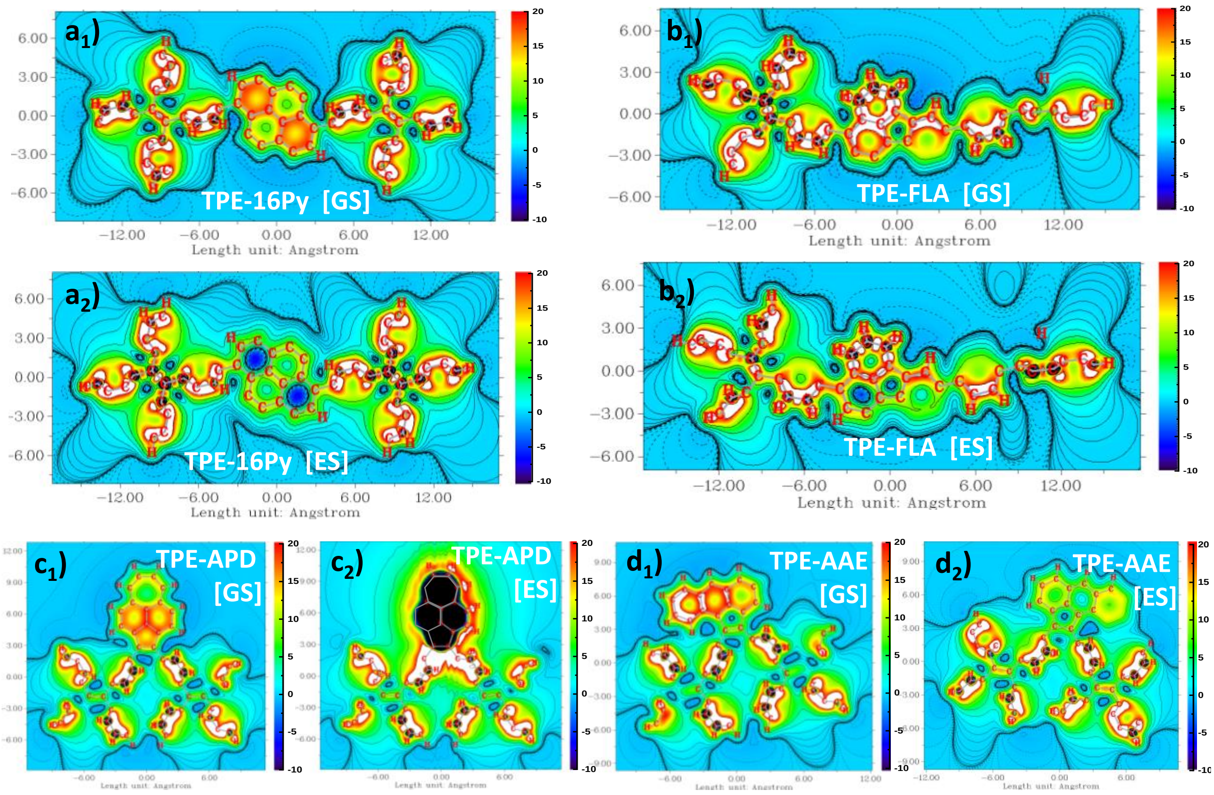

Figure S8. Calculated 2D-ICSS(1)zz maps (1 Å above the XY planes) of the four emitters, a_1)/a_2) TPE-16Py, b_1)/b_2) TPE-FLA, c_1)/c_2) TPE-APD, and d_1)/d_2) TPE-AAE, ground states for a_1)-d_1) and excited states for a_2)-d_2).

Table S3. Calculated key parameters involved in the hole-electron analysis.[a)]

PHAs & excitations		D [Å]	S_r [a.u.]	H [Å]	t [Å]	E_{coul} [eV]	HDI	EDI
	$S_0 \rightarrow S_1$	0.000	0.9182	2.980	-0.827	5.122	6.16	6.18
	$S_0 \rightarrow S_2$	0.000	0.9647	2.918	-0.883	5.319	5.26	5.36
**Py**	$S_0 \rightarrow S_3$	0.000	0.6862	2.713	-1.685	5.493	6.27	7.63
	$S_0 \rightarrow S_4$	0.000	0.9628	2.903	-0.976	5.313	5.18	5.26
	$S_0 \rightarrow S_5$	0.000	0.7107	2.657	-1.622	5.584	7.64	6.21
	$S_0 \rightarrow S_1$	0.790	0.6951	2.695	-1.325	5.555	7.36	6.33
	$S_0 \rightarrow S_2$	0.038	0.8588	2.891	-2.165	5.271	6.27	6.14
**FLA**	$S_0 \rightarrow S_3$	0.034	0.9470	2.657	-1.694	5.618	6.25	5.84
	$S_0 \rightarrow S_4$	0.039	0.9001	2.749	-2.185	5.592	6.29	6.12
	$S_0 \rightarrow S_5$	1.052	0.8673	2.907	-1.196	5.127	5.99	6.20
	$S_0 \rightarrow S_1$	0.403	0.8408	2.896	-1.835	5.258	6.08	6.18
	$S_0 \rightarrow S_2$	0.976	0.7520	2.734	-1.132	5.411	6.29	7.38
**APD**	$S_0 \rightarrow S_3$	0.892	0.7121	2.642	-1.183	5.614	7.95	6.49
	$S_0 \rightarrow S_4$	0.065	0.9490	2.648	-1.650	5.636	5.77	5.92
	$S_0 \rightarrow S_5$	2.108	0.7360	2.673	-0.057	5.173	6.75	6.77
	$S_0 \rightarrow S_1$	0.941	0.6919	2.698	-0.975	5.445	7.70	6.41
	$S_0 \rightarrow S_2$	0.514	0.8043	2.767	-1.600	5.447	6.87	6.40
**AAE**	$S_0 \rightarrow S_3$	0.202	0.9162	2.883	-2.114	5.448	5.95	5.67
	$S_0 \rightarrow S_4$	0.546	0.8714	2.888	-1.776	5.344	6.07	5.80
	$S_0 \rightarrow S_5$	0.359	0.8486	2.824	-1.916	5.444	6.38	6.61
	$S_0 \rightarrow S_1$	0.000	0.5348	2.619	-1.536	5.699	6.77	7.76
	$S_0 \rightarrow S_2$	0.000	0.8845	2.904	-1.797	5.199	6.00	5.89
**DCH**	$S_0 \rightarrow S_3$	0.000	0.7851	2.715	-1.883	5.503	6.24	7.62
	$S_0 \rightarrow S_4$	0.000	0.9657	2.893	-0.804	5.218	5.41	5.35
	$S_0 \rightarrow S_5$	0.000	0.8790	2.758	-1.818	5.448	5.97	5.76
	$S_0 \rightarrow S_1$	0.000	0.7634	2.791	-1.761	5.721	6.18	6.83
	$S_0 \rightarrow S_2$	0.000	0.8548	2.888	-2.206	5.449	6.14	6.41
**DIN**	$S_0 \rightarrow S_3$	0.000	0.62615	3.209	-1.197	5.121	5.74	5.48
	$S_0 \rightarrow S_4$	0.000	0.8495	3.155	-1.753	5.301	5.96	5.43
	$S_0 \rightarrow S_5$	0.000	0.8629	3.165	-1.731	5.278	6.05	5.66

[a)] D, S_r, H and t indices refer to the distance of charge transfer between the hole and the electron, integral of S_r function overlap, function of hole and electron, separation of hole and electron distributions, respectively. E_{coul} refer to the coulomb attractive energy between the hole and electron, HDI and EDI, represent for the hole and electron delocalization index, respectively.

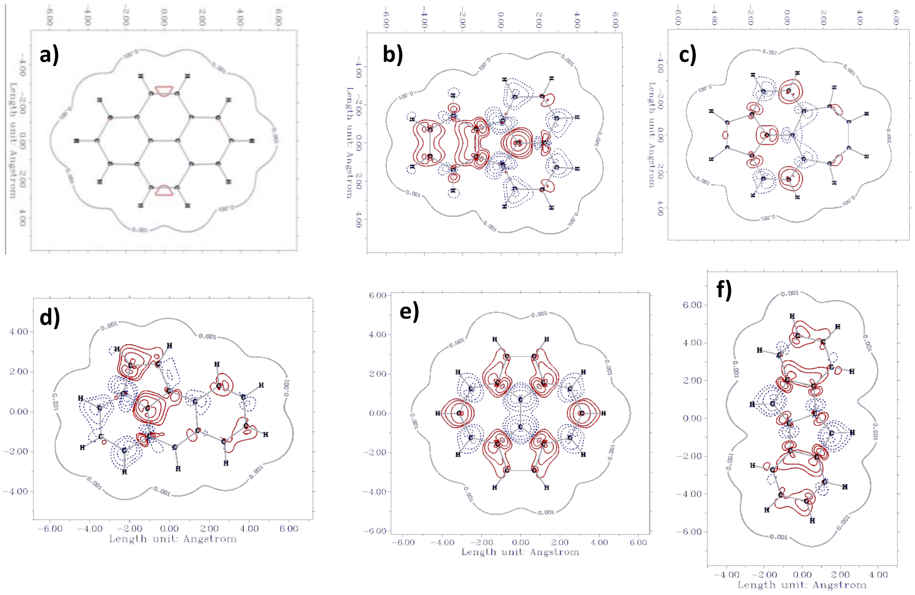

Figure S9. Density difference maps of these PAHs during the electron excitations from ground states to corresponding singlet excited states (red refer to density decrease, blue refer to density increase).

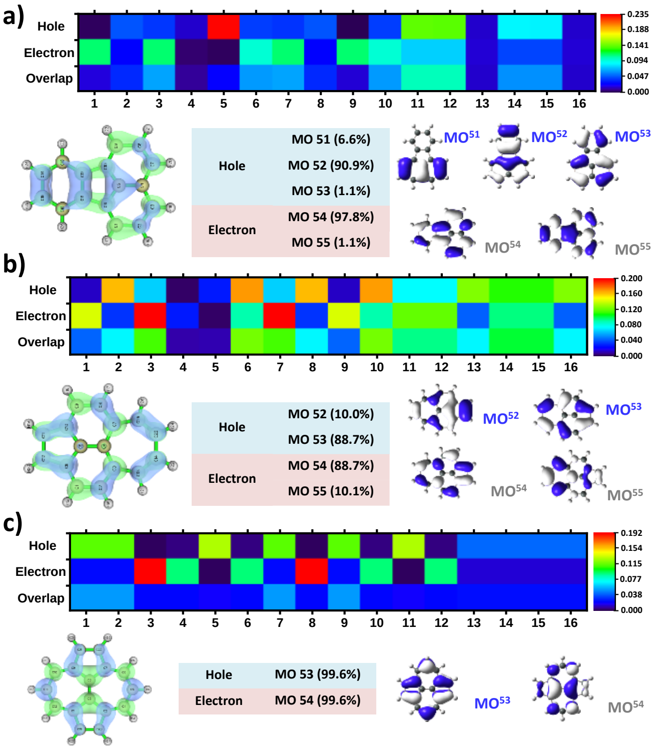

Figure S10. Hole-electron analysis of $S_0 \rightarrow S_1$ transition for a) FLA, b) APD and c) DCH, respectively. With the sequence of atomic contributions to hole and electron in terms of heat map, distribution of hole and electron on molecular skeleton at the same time (green represents the electron distribution, and blue represents the hole distribution), MOs with contribution to hole or electron higher than 1%.

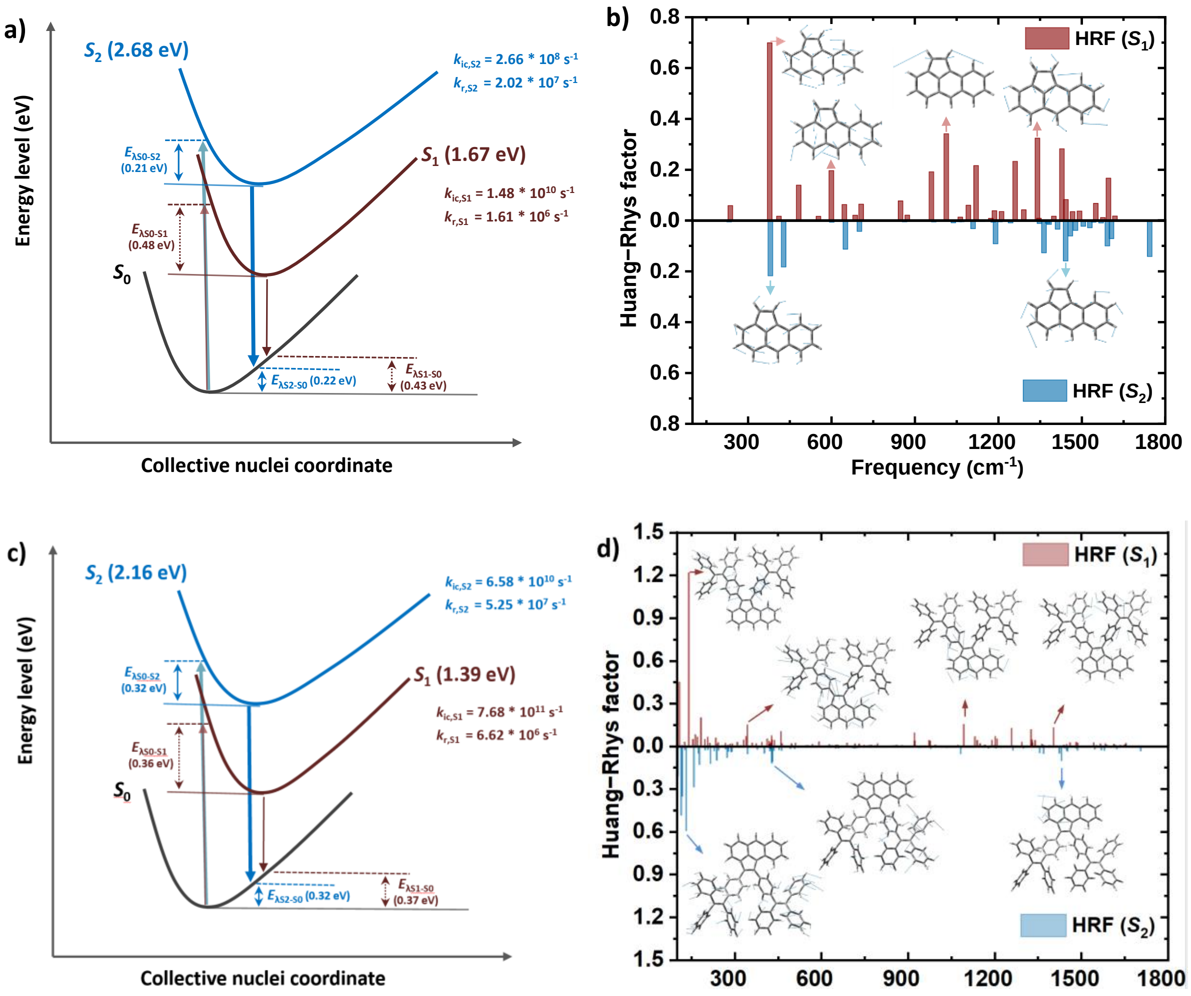

Figure S11. Schematic diagram of the calculated transition rates and reorganization energies of a) AAE/ c) TPE-AAE involved in both S_2 and S_1, and b)/d) the corresponding HRFs versus frequency at various vibration models.

Thermal properties

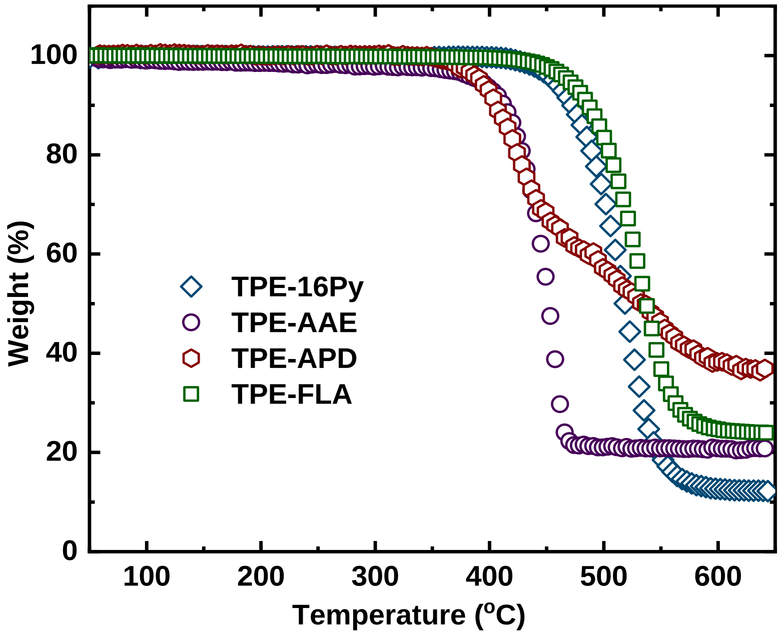

Figure S12. TGA curves of these TPE-modified PAH emitters.

Electrochemical property

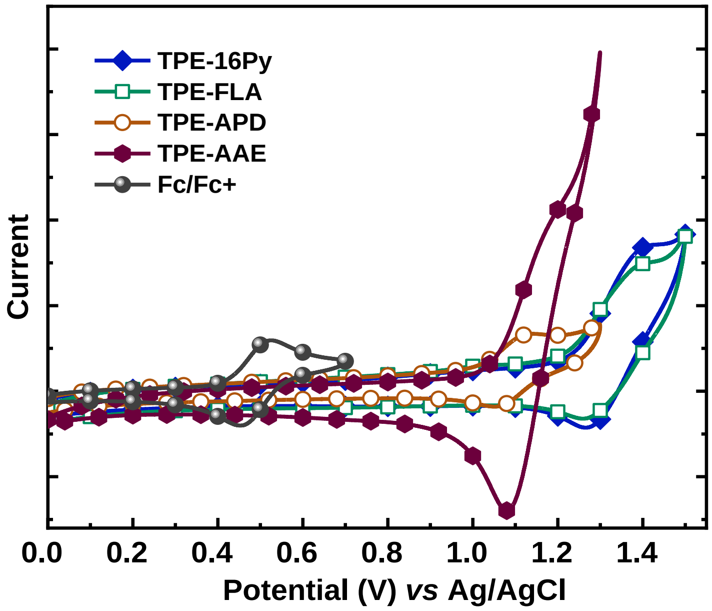

Figure S13. CV curves of these TPE-modified PAHs during the oxidation process.

Photophysical properties

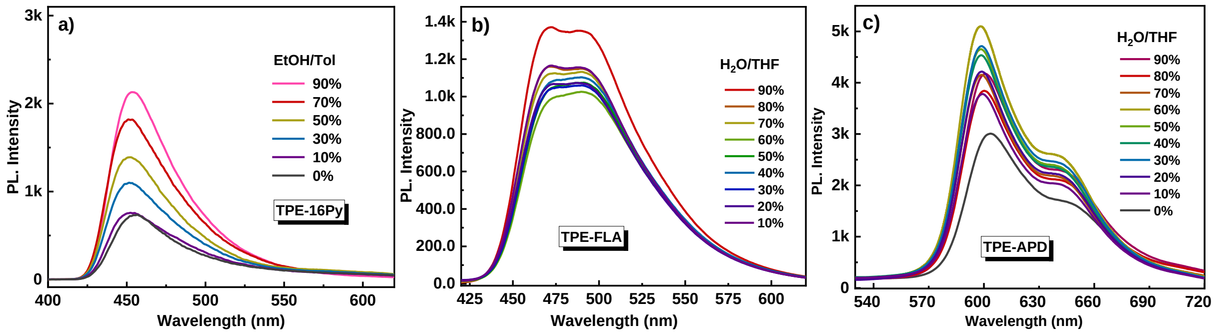

Figure S14. The aggregation-induced emission enhancement (AIEE) feature of a) TPE-16Py, b) TPE-FLA, and c) TPE-APD under the variable volume ratio of H_2O/THF (1×10^{-5} M). Due to the poor solubility of TPE-16Py in THF, the AIEE spectra was recorded in ethanol/toluene instead.

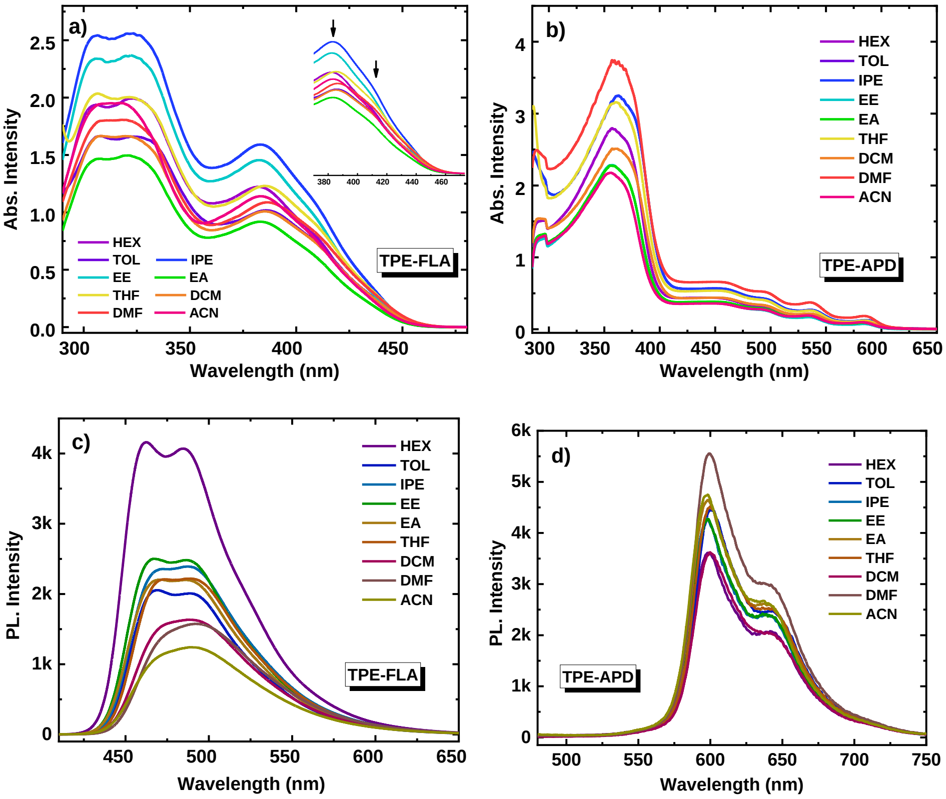

Figure S15. The absorption and photoluminescence spectra of a)/c) TPE-FLA, and b)/d) TPE-APD in different solvents (1×10^{-5} M) with various polarity. Due to the poor solubility of TPE-16Py, the valid spectra in other solvents are not obtained.

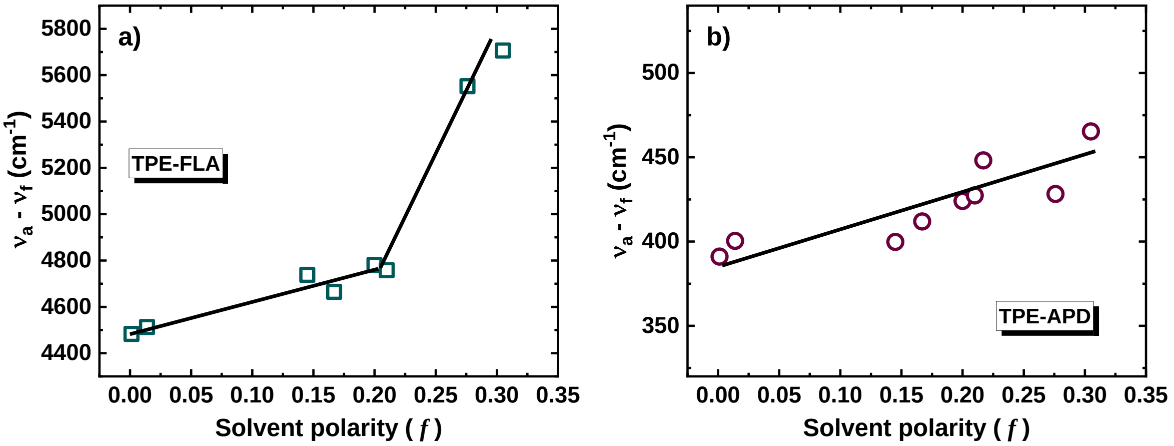

Figure S16. Lippert-Mataga model between Stokes shift (ν_a - ν_f) and solvent polarity (f) of a) TPE-FLA and b) TPE-APD.

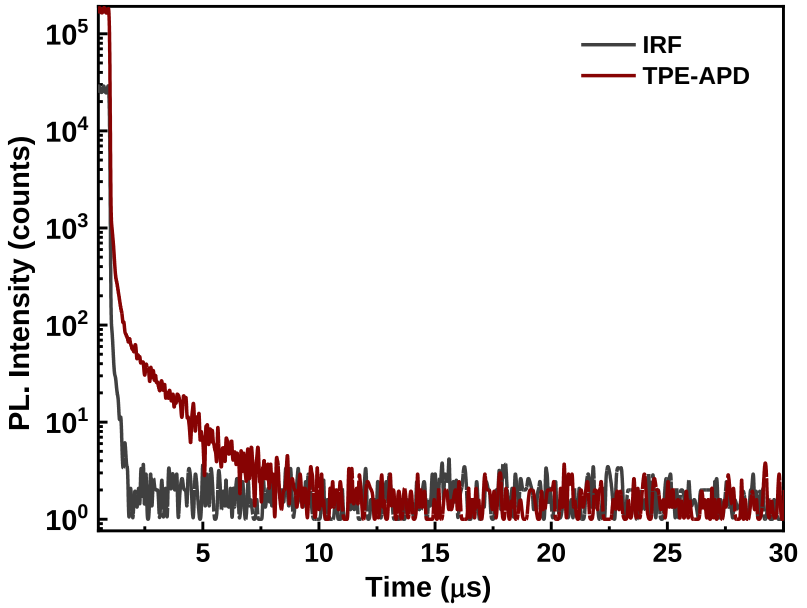

Figure S17. Transient photoluminescence spectra of the TPE-APD in dilute toluene (1 * 10^{-5} M).

Application in organic electroluminescent devices

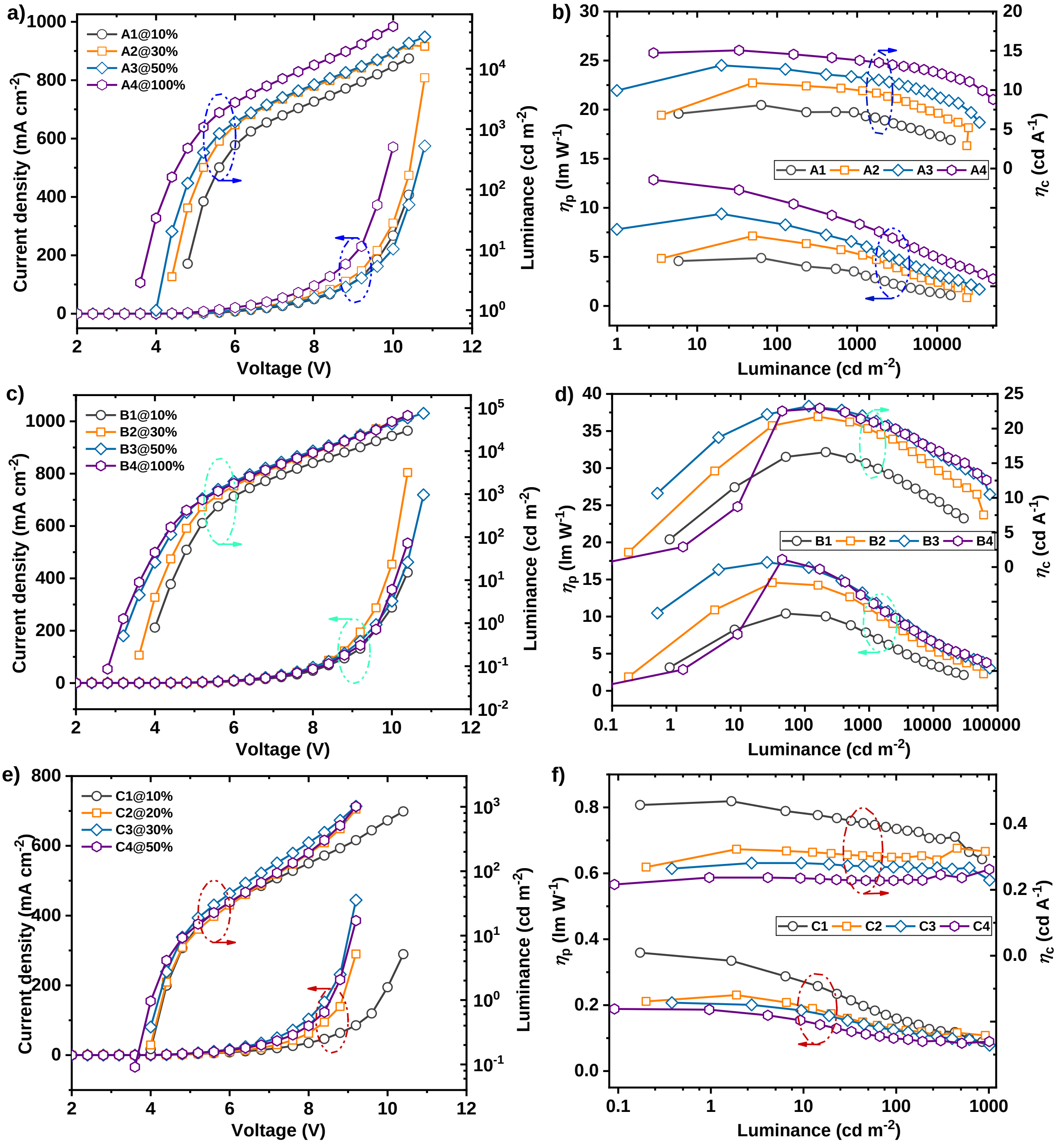

Figure S18. The current density-voltage-luminance (*J*-*V*-*L*) curves, and power efficiency and current efficiency as a function of luminance of these devices, a)/b) for TPE-16Py, c)/d) for TPE-FLA and e)/f) for TPE-APD based devices, respectively.

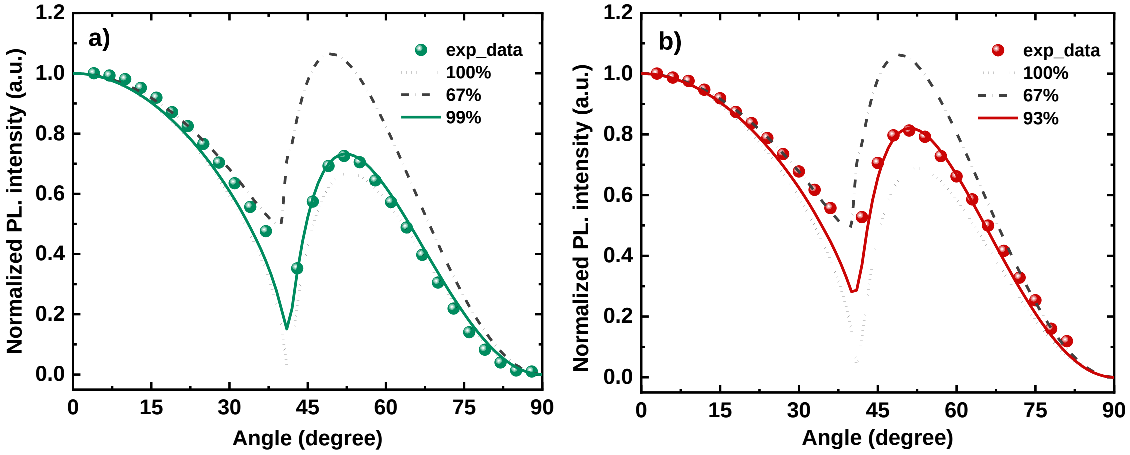

Figure S19. Angle-dependent *p*-polarized PL. intensity and simulation curves for a) TPE-FLA and b) TPE-APD in 26DCzPPy films with 50 wt% doping concentration.

References

[1] G.W.T. M. J. Frisch, H. B. Schlegel, G. E. Scuseria, , J.R.C. M. A. Robb, G. Scalmani, V. Barone, , H.N. G. A. Petersson, X. Li, M. Caricato, A. V. Marenich, et al., *Gaussian 16, Revision A.03* (Gaussian, Inc.: Wallingford CT, 2016)

[2] F. Zhong, X. Yin, J. Wu et al., *J. Mater. Chem. A* ***10*** (2022) 18030-18037.

[3] T. Lu & F. Chen, *J. Comput. Chem.* ***33*** (2012) 580-592.

[4] S. Lin, Q. Ou & Z. Shuai, *ACS Materials Letters* ***4*** (2022) 487-496.

[5] Z. Shuai, *Chin. J. Chem.* ***38*** (2020) 1223-1232.

[6] Z. Shuai & Q. Peng, *Natl. Sci. Rev.* ***4*** (2017) 224-239.

[7] Z. Shuai & Q. Peng, *Phys. Rep.* ***537*** (2014) 123-156.

[8] P. Liu, X.Y. Chen, J. Cao et al., *J. Am. Chem. Soc.* ***143*** (2021) 5314-5318.

[9] Y. Du, L. Wang & K.N. Plunkett, *J. Org. Chem.* ***85*** (2020) 79-84.
